# The process for recognizing of foreign medical degrees in Costa Rica: a statistical survey for the past 15 years

**DOI:** 10.3205/zma001517

**Published:** 2021-11-15

**Authors:** Lizbeth Salazar Sánchez, Juan José Cordero Solis, Alfredo J. López Dávila

**Affiliations:** 1 University of Costa Rica, School of Medicine, San José, Costa Rica; 2 University of Costa Rica, School of Medicine, Recognition Committee, San José, Costa Rica; 3 The German National Institute for State Examinations in Medicine, Pharmacy and Psychotherapy, Mainz, Germany

**Keywords:** medicine, final recognition, Central America, Caribbean, Costa Rica, state examination

## Abstract

This article presents the most important developments in the recognition process of foreign medical degrees in Costa Rica over the past fifteen years. Most applicants received their medical degrees in Cuba, Venezuela, Nicaragua and Mexico. By far the most numerous group completed their studies in Cuba, followed by graduates from Venezuelan and Nicaraguan universities, the number of which has increased in the last five years. The pass rate of the written examination used in the recognition process is 23.9% with relatively large fluctuations between graduates of the individual countries, especially between the countries with the lowest numbers of graduates. The main goal of the recognition process is to ensure that graduates from different study conditions and curricula as well as from diverging areas of specialization of the faculties abroad have competencies and knowledge comparable to those of medical graduates in Costa Rica. The focus is on the safety of the patient, as is the case with state exams in many countries.

## Zusammenfassung

Der vorliegende Beitrag präsentiert die wichtigsten Entwicklungen des Anerkennungsprozesses der im Ausland erworbenen medizinischen Abschlüsse der letzten fünfzehn Jahre in Costa Rica. Die meisten Bewerber haben ihren Medizin-Abschluss in Kuba, Venezuela, Nicaragua und Mexiko erworben. Die mit Abstand zahlreichste Gruppe hat ihr Studium in Kuba absolviert gefolgt von Absolventen von venezolanischen und nikaraguanischen Universitäten, deren Anzahl in den letzten fünf Jahren zugenommen hat. Die Bestehensquote der im Rahmen des Anerkennungsprozesses angewandten schriftlichen Prüfung beträgt 23,9% mit relativ großen Schwankungen zwischen Absolventen der einzelnen Länder, besonders denjenigen mit den geringsten Anzahlen Absolventen. Das Hauptziel des Anerkennungsprozesses ist es zu gewährleisten, dass Absolventen aus verschiedenen Studienbedingungen und Curricula sowie aus divergierenden Schwerpunkten der Fakultäten im Ausland vergleichbare Kompetenzen und Kenntnisse aufweisen, die mit jenen der in Costa Rica graduierten Medizinern vergleichbar sind. Im Vordergrund steht dabei die Sicherheit der Patienten, wie es auch bei Staatsexamina in vielen Ländern der Fall ist.

## Introduction

The international migration of physicians and other health care graduates had been a worldwide phenomenon long before the advent of the digital age and its associated facilitation of communication and mobility. More than forty years ago, it was reported that countries graduating more physicians than they can financially support become emitters of this highly skilled workforce. Conversely, those who produce fewer physicians than they can reasonably afford tend to become recipients. Even though other causes of migration have been identified (e.g. working conditions), these can be seen as derived from the main factor or as secondary to economic reasons [1]. For detailed descriptions see [2], [3]. With regard to the mentioned economic factor, the countries of the OECD organization can be examined as a model in order to characterize the global migration of medical professionals as uncomplicatedly but simultaneously as representatively as possible. Being economically particularly strong, some countries of this organization are a very frequent target for immigrants and at the same time provide reliable data. For instance, a total of 55% of all foreign-born or foreign-trained doctors practicing medicine in OECD countries have established themselves in the United States of America (USA) and the United Kingdom (UK) (42% and 13% in the USA and UK, respectively). Due to this considerable immigration, the mentioned nations represent a large data pool. If those who had studied before their emigration are isolated from this data pool, two levels of migration of graduates can clearly be identified: a first level can be classified as “global”, with persons traveling a very long distance around the globe to establish themselves. India and Pakistan are the main source of these already trained medical doctors. Together, these two countries bring 43% and 27% of all overseas graduated medical doctors practicing in the UK and US, respectively. An additional “regional” level can also clearly be identified, with persons staying much closer to their country of origin. Around 15% of all abroad-trained doctors practicing in the UK come from Europe, while around 10% of all abroad-trained doctors practicing in the USA come from Canada, Mexico or the Dominican Republic [4]. This regional migration is also largely attributable to an economic motivation. For instance, better salaries and training in the destination country as well as poor professional prospects in the country of origin have been reported as the main reasons for the migration of medical doctors within the European Union [5], [6], [7]. Central America and the Caribbean are also experiencing migration of medical doctors, and Costa Rica is a frequent destination for graduates from abroad [8]. Thus, examining data from this country, which has recently also been accepted into the OECD organization, enables a further analysis of the migration of doctors from a complementary perspective.

### Recognition process

Recognition of medical degrees by the University of Costa Rica (UCR, the main state university in the country) is a condition set out in the law of the Colegio de Médicos y Cirujanos de Costa Rica (G-CMC) [http://www.pgrweb.go.cr/scij/Busqueda/Normativa/Normas/nrm_texto_completo.aspx?param1=NRTC&nValor1=1&nValor2=90625] in order to allow doctors with a foreign degree to practice in Costa Rica. The “Colegio de Médicos y Cirujanos de Costa Rica” corresponds to the Federal Medical Association in Germany and is therefore referred to as the Medical Association from here on. Only those whose titles are recognized by the UCR may join the Medical Association and practice the profession. This process is regulated by the “Ordinance on the Recognition and Equalization of Qualifications of Other Higher Education Institutions” (VAA-UCR) [https://www.cu.ucr.ac.cr/normativ/reconocimiento.pdf], whose definitions for each term are based on the “Agreement on the Nomenclature of Degrees and Titles of State Higher Education” (AN-UCR) [https://www.cu.ucr.ac.cr/normativ/nomenclatura_grados_titulos.pdf] and the “Ordinance of Article 30 of the Agreement on Coordination of State Higher Education” (AK-UCR) [https://www.cu.ucr.ac.cr/uploads/tx_ucruniversitycouncildatabases/normative/articulo_30_conare.pdf]. Simplifying, the G-CMC is a law of the Republic of Costa Rica that assigns the UCR the process of recognizing medical degrees obtained abroad. The VAA-UCR, AN-UCR and AK-UCR are the institutional regulations used for this purpose. This means that the recognition process is administered by the UCR, yet it has state character. The process begins with a formal application by the candidate before the Office for University Planning of the National Rectors' Council, an alliance to which all state universities in the country, including the UCR, belong. In addition to personal data, the application contains:

1. the curriculum and the medical diploma of the foreign institution,
2. a certificate of good conduct or its equivalent, issued by the country of origin or country of residence,
3. official documents certifying medicine practice in the country of origin or country of residence for the last two years,
4. official documents on the study plan of the clinical training and its approbation during the last year of study (practical year or equivalent), which must include rotations of at least ten weeks in the following medical specialties: gynecology and obstetrics, internal medicine, pediatrics, surgery and community health.

Clinical training must include a hands-on component of at least 80% of the time as well as night shifts. All documents must be duly certified by the authorities in the country of origin of the documentation, if necessary with an official translation into Spanish. After the corresponding review, the documents are sent to the “Escuela de Medicina” (Medical Faculty of the University of Costa Rica. EM-UCR), where an academic committee (so-called recognition committee) is convened to give an assessment. This shows whether the applicant is entitled to take part in a recognition test. For this purpose, all admitted applicants are convened for a written examination once a year. The examination evaluates clinical as well as preclinical knowledge. Applicants who pass the exam receive a diploma for the recognition of their degree, which is accepted by the Medical Association.

#### Aims of the study

The aims of this article are:

1. to present the results of the recognition process of foreign medical degrees in Costa Rica over the past fifteen years,
2. to briefly discuss possible factors that could explain these results and
3. to characterize migration of medical doctors to Costa Rica in a global and regional context.

Our analysis also enables general comparisons between applicants with foreign degrees and national medical students in standardized written tests of medical knowledge.

## Methods

The study consists of a descriptive-retrospective analysis of the recognition process for medical degrees obtained abroad administered by the UCR between 2005 and 2019. Presented information includes the country in which the applicants studied (which does not necessarily correspond to their nationality) as well as the outcome of the process. The most relevant data is presented using descriptive statistics. The information originates from the files of the EM-UCR Recognition Committee and is handled and presented anonymously. All original data is provided in tabular form as supplementary material (see attachment 1 ).

## Results

**Figure 1:**
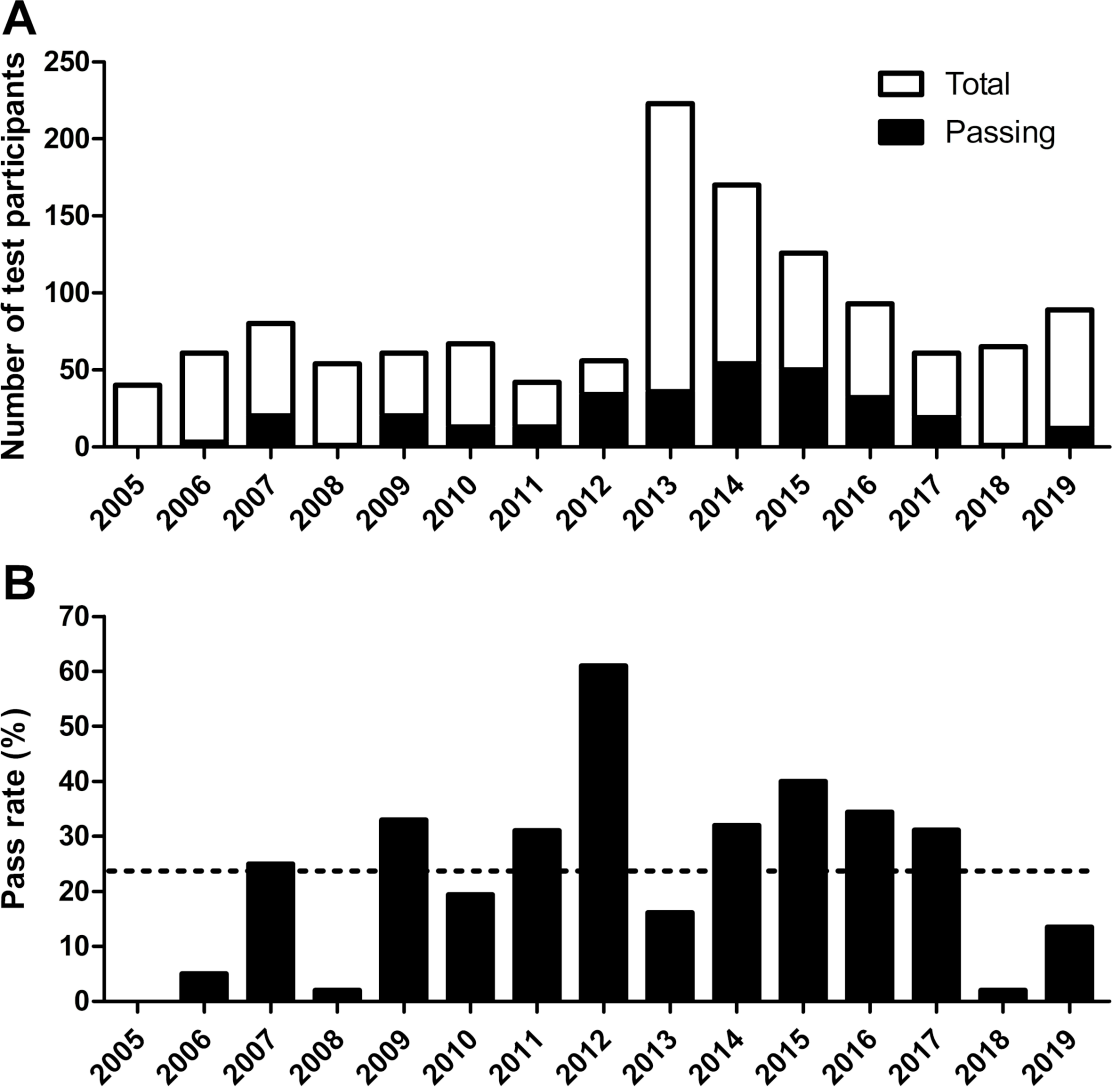
Total number of test participants in the years 2005 to 2019 and their pass rate. A. Total and passing number of test participants. B. Percentage of passing participants normalized to their total number. The dotted line shows the mean pass rate (23.9%). n=1288 examinees.

**Figure 2:**
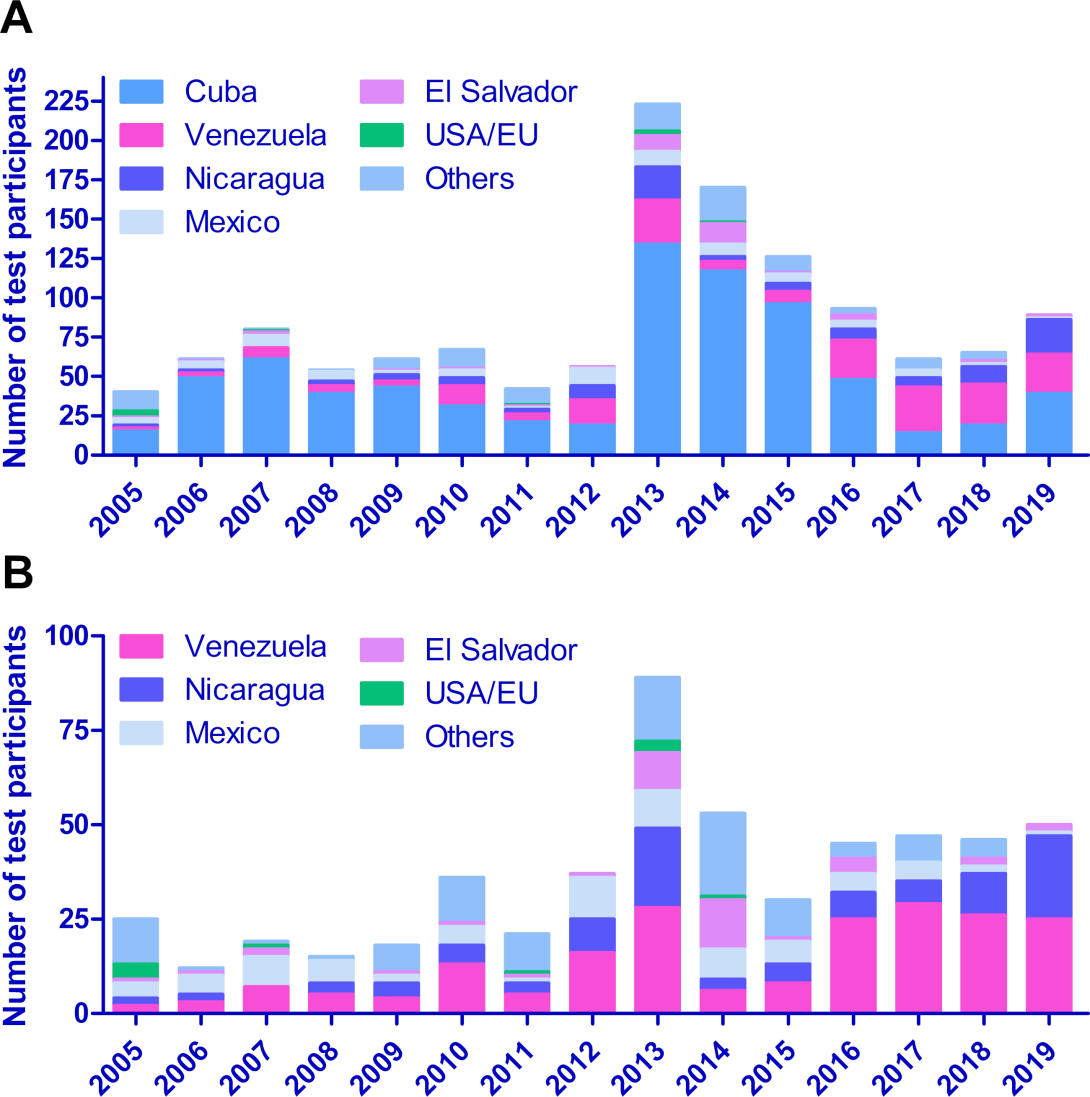
Total number of test participants per year, disaggregated according to the country of origin of their degree. A. For the period 2005-2019, the candidates graduating in Cuba were by far the largest group. B. After subtracting the group Cuba, it becomes clearer that candidates from Venezuela and Nicaragua were the second and third largest groups and that their number has increased over the past five years. USA: United States of America. EU: Europe. n=1288 examinees.

Between 2005 and 2019, the EM-UCR recognition committee admitted a total of 1881 applicants into the recognition test of medicine. Of the admitted persons, 1288 (68.5%) took the exam and only 308 of them (23.9%) passed it. Figure 1 (Fig. 1) describes the number of test participants per year, as well as their corresponding pass rate in percent. 2012 was the year in which the highest pass rate was achieved (61%), followed by 2015 (40%). In contrast, no one passed in 2005. Figure 2 (Fig. 2) shows the breakdown of candidates by country of origin of their degree, which clearly shows that most of them graduated in Cuba.

The decreasing number of test participants over the past six years is strongly influenced by the decrease in the group Cuba (see figure 1 (Fig. 1), point A and figure 2 (Fig. 2), point A). This in turn is partly explained by the fact that many Costa Ricans have studied medicine in Cuba in the past. However, this subgroup (Costa Ricans with Cuban degree) has decreased considerably over the past five to six years. Since the largest group Cuba also includes these returning Costa Ricans, and these are much less common in other groups, it makes sense to subtract the group Cuba from the total volume in order to get a better overview of the actual immigration of doctors in the region. This adjustment makes it clear that in the last fifteen years an increasing number of foreign medical doctors have tried to obtain a title recognition in Costa Rica (see figure 2 (Fig. 2), point B). Graduates from Venezuela and Nicaragua, the number of which has increased continuously in recent years, accounted for the largest share of growth and represented the second and third largest group of test participants. The increasing number of candidates from Venezuela and Nicaragua is associated with a corresponding increase in the number of applications of graduates from these two countries.

**Figure 3:**
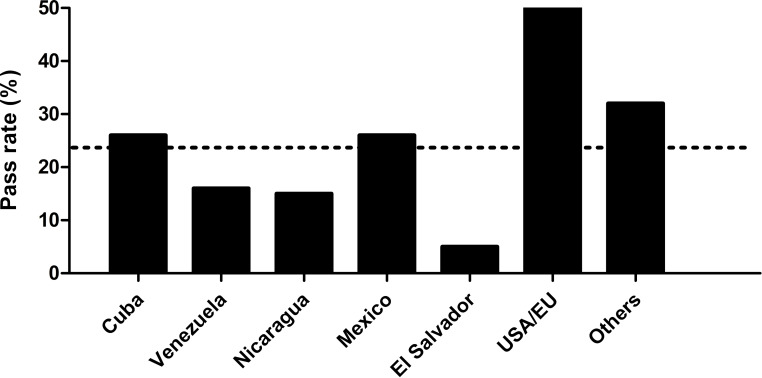
Pass rate of all test participants disaggregated by country of origin of their degree. The dotted line shows the mean pass rate. The groups are ranked in descending order according to the number of examinees. An exception is the group “Others”, whose total number is not the lowest, yet is made up of a very small number of participants from numerous countries. USA: United States of America. EU: Europe. n=1288 examinees.

Finally, figure 3 (Fig. 3) presents the percentage of test participants passing the test in the timeframe mentioned above, disaggregated by country. The figure shows that the groups with the most candidates (Cuba, Venezuela, Nicaragua and Mexico) achieved a pass rate between ~15% and ~25%. On the other hand, the groups with the lowest number of test participants showed a greater variation, with extreme pass rates of ~5% for test participants from El Salvador and ~50% for test participants from the United States of America (USA) and Europe (EU). This last group is by far the smallest and consists of only 10 persons. The “Others” group is made up of persons from different countries, yet the most of them come from Latin America. Nations such as Colombia, Paraguay, Honduras, Brazil and the Dominican Republic are represented here most frequently (see the supplementary material including the number of examinees per year by country of origin, see attachment 1 ).

## Discussion

The present study portrays the results of the recognition process of medical degrees acquired abroad over the past fifteen years in Costa Rica. The main result is undoubtedly the high participation of applicants who have studied in Cuba, Venezuela, Nicaragua and Mexico, as well as the overall low pass rate of the recognition test 23.9% on average. This could be even lower if applicants were admitted to the recognition test without prior verification of their academic background.

Considering the number of examinees by country of origin makes immediately clear that most of them come from the immediate geographical proximity of Costa Rica, namely from Latin America with Central America and the Caribbean as the front runners. The big global players India and Pakistan are not represented. Applicants from Spain cannot be found either, even though they speak the same language. From these observations it can be determined that Costa Rica experiences predominantly regional immigration o medical doctors. The increasing number of applications for the recognition of medical degrees obtained abroad in Costa Rica is a trend that has been documented not just since 2005, but since the end of the 20th century. In 1990, for example, 44 applications were submitted, whereas in 1998 these had already risen to 188, corresponding to a four-fold increase in the number of applicants [8]. During this period, most of the applicants had already completed their medical studies in Cuba (followed by Mexico at the time), as has been the case for the past fifteen years. In the last decade of the 20th century there were no applications for recognition of medical degrees from Venezuela [8]. However, the number of applicants who have studied in this country has increased considerably since 2005, so that it is currently the second largest group of participants in the recognition test (see figure 2 (Fig. 2), point B). The number of applicants from Nicaragua has also increased significantly since 2018. It is noticeable here that in both countries the increase in the number of applicants coincides with phases of political and economic instability. Although our data do not reveal a causal relationship, it is known that the economic and political situation of a country are important factors in the emigration of medical doctors and of persons as a whole [9], [10], [11]. Costa Rica would thus benefit from the regional situation, in this specific case the immigration of doctors who integrate into the health system. 

Although the available data are not sufficient to clarify categorically the causes of the rather low pass rate of the recognition test, possible influencing factors arise for discussion. As part of the recognition process, for example, no applicant is favored or disadvantaged because of their language in order to be admitted to the Spanish-language recognition test. However, almost all applicants speak Spanish fluently or even as their mother tongue, as they mostly come from Latin America or at least have completed their medical studies in the region. A language barrier can therefore be excluded as an explanation of the low pass rate, especially since this would only affect a very small number of applicants, for example a few from the groups USA/EU and Others.

Graduates from Cuba, Venezuela, Nicaragua and Mexico together make up 87.6% of all test participants. This main group has pass rates between ~15% and ~25% and thus a relatively small spread. On the other hand, graduates from El Salvador and the USA/EU make together only 3.9% of all test participants, which could explain the very high dispersion in these groups. This is supported by the fact that the group “Others”, with 109 examinees (comparable to Nicaragua), is considerably closer to the mean than the USA/EU group, which consists of only 10 examinees. The reason for the strong variation is therefore not necessarily a generalizable higher performance of the graduates from the USA/EU, or a worse performance of the graduates from El Salvador. An alternative, equally plausible explanation of the variation is, however, the fact that the graduates of the USA/EU group completed their studies in universities that are usually well above the position of Central American and Caribbean universities in the international university rankings. As a result, the higher pass rate of this group could actually be explained by better preparation through their studies.

In the past, the overall low pass rate was a reason to contest the result of the recognition test on the grounds that it would be of limited quality and would not evaluate the applicants’ performance in an appropriate manner. In this context, it should be mentioned that the UCR classifies the test as a “test with significant consequences”. This means that the institution is aware of the particular importance of the process from the applicant's point of view, and that special attention is paid to its correct preparation and implementation through several control mechanisms. In this sense, the exam is not only subjected to a philological examination, but also reviewed by experts in the design of cognitive tests and subjected to pilot studies and validation processes prior to their application. A detailed explanation of these measures is outside the scope of this article, but could be the subject of a future publication.

The aim of the recognition process as well as the implementation of the written examination is by no means to prevent the practice of medicine in Costa Rica for graduates from abroad, but to ensure the health of the patients and the quality of their health care. It has to be ensured that those who practice medicine in the country possess the necessary range of skills and knowledge. This goal is also pursued through state examinations in various countries: “Especially when study conditions and curricula are different and possibly also reflect diverging focus areas of the faculties, for example in model study courses, it is important that career starters have comparable skills regardless of their place of study and ideally regardless of the country of training. This safety for patients can only be achieved through standardized tests” [12]. In line with this endeavor, another goal of the recognition process is that graduates from abroad achieve a similar level of performance as local medical graduates. This guideline also largely explains the prerequisites mentioned in the introduction for being admitted to the recognition test. For example, the length of the practical year and its five mandatory subject areas are requirements that also have to be met by those studying medicine in Costa Rica.

With more than 40,000 active students, a significant volume of research production and more than 280 international academic agreements, the UCR is positioned in the 531-540 range of the global “QS-World University Rankings” for 2022 [https://www.topuniversities.com/university-rankings/world-university-rankings/2022] and ranks 20^th^ among all Latin American Universities as well as on the first position in Central America [https://www.topuniversities.com/university-rankings/world-university-rankings/2021]. In line with this strong regional positioning, the EM-UCR students are very competitive. For the past four years, the National Board of Medical Examiners’ International Foundations of Medicine (IFOM, Clinical Science Exam. NBME) exam has taken place in the country to gain access to the practical year. The EM-UCR students received consistently high pass rates in this test (between 94% and 98%) and thus not only surpassed the national (including private universities that also offer medical degrees), but also the international average. The achievement of many of these students is even recognised every year by the NBME with individualized diplomas. From this, it could be deduced that an examination formulated by the EM-UCR (as is the case for the recognition process) presupposes a relatively high standard for the region. For instance, medical graduates of the EM-UCR, play a key role in the preparation of the recognition test, as they are subjected to it as part of the pilot studies and validation processes.

Based on the above discussed issues, it can at least be assumed that a difference between the preparatory profile of the applicants and the requirements profile of the test could be a key aspect that explains the low pass rate of the recognition test. The preparatory profile of the applicants should not be reduced to the individual learning before the written examination, but also depends on other background factors such as the country and university of the degree, curriculum and focus of the study as well as practical experience. These factors are largely adjusted through the selection process for admission to the examination. The requirements profile of the examination results from its pilot studies and validation processes, as well as from the overarching goal of ensuring the highest possible patient safety, as is the case with the EM-UCR students. In order to improve the candidates' chances of passing, options are currently being considered in order to better adapt their preparation profile to the requirements profile of the test. For instance, training strategies and access to bibliographic resources are conceivable. The further development of the recognition process including the examination, the profile of the applicants (country of completion, number, etc.) as well as the results (pass rate) could well be matter of a future report.

## Outlook and commentary on the COVID-19 pandemic

For 2020 there were approximately 50 persons interested in obtaining a recognition of their medical degree in Costa Rica. However, the COVID-19 pandemic and the hygiene measures taken by the health authorities to protect the population have significantly restricted the process in the last year (meeting of the recognition committee, preparation of the exam, etc.), so that the examination has not been called in 2020. A replacement test has been carried out in March 2021. However, due to successive waves of infections and border closings in several countries in the region only a fraction of those admitted attended the call. A next test in November 2021 has already been called, with a very heterogeneous and in general, a slow vaccination rate in Latin America and the resulting varied consequences as the main challenge for the coming development. The COVID-19 pandemic has left devastating economic and social consequences in Latin America, which will certainly be reflected in the regional and global migration of medical doctors in the years to come.

## Conclusion

Costa Rica is an attractive country for medical graduates from the region. Most applicants for a title recognition have studied in Cuba, Venezuela, Nicaragua and Mexico. The passing rate of the written examination for title recognition has been ~24% over the past 15 years.

## Acknowledgements

The authors would like to thank Ms. S. Bermúdez, employee of the EM-UCR, for administrative support, collection and processing of information. The authors would also like to thank Ms. S. López for her valuable comments on the manuscript.

## Competing interests

The authors declare that they have no competing interests. 

## Supplementary Material

supplementary material
