# Supplementary material for: The process for recognizing of foreign medical degrees in Costa Rica: a statistical survey for the past 15 years
Source: GMS J Med Educ. 2021 Nov 15;38(7):Doc121. doi: 10.3205/zma001517 (PMC8675375; doi:10.3205/zma001517)
Supplement: supplementary material [file JME-38-7-121-s-001.pdf]

Attachment 1: Supplementary material

Table 1: Total number of test participants per year, disaggregated according to the country of origin of their degree, pass and absenteeism rates.

Year	Ad	Ta	Cu	Ve	Ni	Me	ES	U/E	O	Pa	PR (%)	AR (%)
2005	46	40	15	2	2	4	1	4	12	0	0,0	13,0
2006	80	61	49	3	2	5	1	0	1	3	4,9	23,8
2007	102	80	61	7	0	8	2	1	1	20	25,0	21,6
2008	59	54	39	5	3	6	0	0	1	1	1,8	8,5
2009	61	61	43	4	4	2	1	0	7	20	32,8	0,0
2010	95	67	31	13	5	5	1	0	12	13	19,4	29,5
2011	53	42	21	5	3	1	1	1	10	13	30,9	20,8
2012	106	56	19	16	9	11	1	0	0	34	60,7	47,2
2013	253	223	134	28	21	10	10	3	17	36	16,1	11,9
2014	388	170	117	6	3	8	13	1	22	54	31,7	56,2
2015	285	126	96	8	5	6	1	0	10	50	39,7	55,8
2016	100	93	48	25	7	5	4	0	4	32	34,4	7,0
2017	78	61	14	29	6	5	0	0	7	19	31,1	21,8
2018	83	65	19	26	11	2	2	0	5	1	1,5	21,7
2019	92	89	39	25	22	1	2	0	0	12	13,5	3,3
**Total**	**1881**	**1288**	**745**	**202**	**103**	**79**	**40**	**10**	**109**	**308**	**23,9**	**31,5**

Ad: admitted to the test, Ta: taking the test, Cu: Cuba, Ve: Venezuela, Ni: Nicaragua, Me: Mexico, ES: El Salvador, U/E: USA and Europe, O: Others, Pa: passing the test, PR (%): percentual pass rate, AR (%): percentual absenteeism rate.

Table 2: Pass rate of all test participants disaggregated by country of origin of their degree.

Country	Number of test participants	Number of passing participants	Pass rate (%)
Cuba	745	195	26,2
Venezuela	202	34	16,8
Mexico	79	21	26,6
Nicaragua	103	16	15,5
El Salvador	40	2	5,0
USA und Europa	10	5	50,0
Others	109	35	32,1
**Total**	**1288**	**308**	**23,9**
